# Bio-Politics and Calculative Technologies in COVID-19 Governance: Reflections From England

**DOI:** 10.34172/ijhpm.2021.134

**Published:** 2021-09-20

**Authors:** Kelum Jayasinghe, Tarosha Jayasinghe, Chaminda Wijethilake, Pawan Adhikari

**Affiliations:** ^1^Faculty of Social Sciences, University of Essex, Colchester, UK.; ^2^Norwich Medical School, University of East Anglia, Norwich, UK.

**Keywords:** COVID-19, Calculative Technologies, Bio-Politics, Technologies of Government, Self-governance

## Abstract

**Background:** Through the extensive use of public media, the government of England was heavily involved in encouraging and instructing people on how to manage their life during coronavirus disease 2019 (COVID-19). This model of health emergency governance replicates the practice of ‘calculative technologies’ and ‘bio-politics’ embedded in population management. Previous research on COVID-19 governance both in the United Kingdom and beyond provides varied revelations on broader ‘technologies of government’ and bio-politics by numerous governments. However, rarely have any studies explicitly and distinctively highlighted the unique ‘calculative technologies’ mobilised by governments within their bio-politically designed "technologies of government" to compel the populations to manage their lives under their COVID-19 guidance. The paper therefor examines how the UK government deployed "calculative technologies," as part of its strategies of health governance and governmentality during the first wave of COVID-19 in England.

**Methods:** This study uses document analysis as its data collection method. Its review includes documents, press releases, social media disclosures and health guidance issued by the UK government from March to December, 2020. The data are analysed employing the Foucault’s governmentality and bio-political scholarship.

**Results:** The paper’s findings reveal the UK government’s use of integrated calculative technologies of self-governance in the form of risk calculations and metrices/statistics (eg, death tolls, infection rates), performance management (eg, two metre social distancing, and hand washing for twenty seconds) and discipline and control (eg, fourteen days self-isolation), in addition to a more conventional top-down, managerial decision-making process adopted in the past. By these newly initiated "calculative technologies," the government has "bio-politically" governed the behaviours and lifestyles of vulnerable community members, health workers and general public at a distance, inculcating self-management and individualisation of responsibility.

**Conclusion:** The newly adopted calculative technologies used by the UK government created a multi-faceted discourse of obligations, entitlements and scale of engagement, and facilitated directions about what people should do to protect themselves and others from the spread of the virus. Overall, the overtly and idiosyncratically used calculative technologies resemble a unique ‘art of government’ and produce a set of ‘bio-political’ interventions enforcing the populations to manage their own wellbeing and governing them at a distance during COVID-19.

## Background

**Key Messages**

** Implications for policy makers**

- Calculative accounts/matrices, technologies and instruments play an important role in the politics of daily life, and they made the government’s health emergency governance discourse visible and measurable for the public, particularly during the pandemic.
- Those governing throughout health crises should endeavour to enact better ‘health and recovery’ rather than overly using ‘self’ governance strategies to politically control the population.
- It is important to establish specific bio-political techniques, instruments and policies to target vulnerable communities, ie, ethnic minority groups, in order that they are ‘equitably’ treated during the pandemic.

** Implications for the public**

With decades of neo-liberal encroachment through policy reforms and austerity measures, a succession of UK governments has allocated inadequate funding and investment in the National Health Service (NHS). This has made them unprepared for high-impact healthcare emergency situations such as that created by coronavirus disease 2019 (COVID-19). The constructively built ‘bio-policies’ with tailor made calculative technologies, including instruments and metrices, as well as greater awareness and advocacy, can help citizens, particularly vulnerable people living with inherited life risks, to gain better protection and life expectancy as equal citizens of society during such emergency situations. However, the vulnerable populations and disadvantaged groups, ie, ethnic minority backgrounds (BAME – Black, Asian, and Minority Ethnic), have been further marginalized and are facing increased obstacles in terms of receiving the necessary care and treatment due to the application of “calculative technologies” to shape health behaviours.

This paper examines how the UK government deployed calculative technologies to govern citizens during the first wave of COVID-19 in England. The previous researches on governmentality and the bio-politics of COVID-19 in the United Kingdom and other countries *ignore* the importance of calculative technologies in population governance during a pandemic, as elaborated in this paper. Previous studies on COVID-19 governance in the United Kingdom^1-4^ *limit* their focus toward the managerial and decision-making effectiveness of the UK government, including preparedness and responses, crisis management, performance communication and accounting and accountability, and overlook the explicit and distinctive connection between calculative technologies and bio-political aspects of population governance. For example, Joyce^2^ examines the preparedness for the pandemic in respect of the capacity for surveillance, governance and coordination structures and the modes of crisis management used by the UK government. Ahmad et al^1^ explore the UK government’s strategies executed for governing the population at a distance, specifically the COVID-19 testing policies and underlying policy rationale. Their analysis merely reveals how the testing targets were set, operationalized and used as a performance communication mechanism. Moreover, Ahrens and Ferry^3^ use the Foucauldian notion of ‘normalisation’ to reveal the role of accounting, eg, monthly statistical reporting, financial reporting and budgeting flexibilities in establishing the operational accountability of the government regarding regularity, probity, value for money and fairness. All these studies reveal some calculative technologies used by the UK government but ended up focussing merely on managerial effectiveness rather than bio-political implications. On the other hand, by analysing the stories of the COVID-19 public health catastrophe (the death of over 57 600 and C19 death certificates), and the public relations exercise by the UK government, Morgan^4^ reflects how culture (in the form of figurative expression) is able to directly impinge on political process, public behaviour, and control over the spread of the virus, but misses its link with calculative technologies.

 Similar researches beyond the United Kingdom also* neglect the *importance of explicit and distinguishing calculative technologies in governing populations. Instead their works largely refer to broader governance technologies, such as a complex set of ‘liberal’ and/or ‘coercive’ technologies that are used to rationalise the political power of governments.^5-10^ For instance, through the genealogical analysis of COVID-19 bio-politics, Marinković and Major^9^ recognize first, the transformation of the old biological regime and the emergence of the ‘gaze’ as a technology of power/knowledge, and then the emergence of biopolitical power over life and the central problem of instability in a ‘new normalcy’ without explicit reference to specific calculative technologies. Applying a triad of concepts – sovereignty, governmentality, and post-liberalism analysis of a corona-imposed state of emergency in Estonia and Finland – Makarychev and Romashko^5^ report how biopolitical anti-crisis management techniques are used to persuade people to sacrifice personal liberties for the sake of public safety. Closer to the current study, Gjerde^6,7^ utilizes the governmentality studies and a Foucauldian discourse analysis to report the Norwegian government’s two responses to COVID-19, initially the articulation of a liberal rationality and later replaced by an interventionist bio-political approach that restricts freedoms and economic progress in favour of safeguarding the health of the population. Gjerde^7^ specifically uncovers the mentalities and broader technologies of power employed by the Norwegian government as it attempts to control the COVID-19 pandemic. Moreover, Giritli Nygren and Olofsson^8^ show how the normally risk averse and cautious Swedish government employed what some considered a soft and irresponsible approach during the pandemic with a greater exercise of power and authority, eg, disciplining and policing,^11-14^ rather than definite risk management strategies. However, their policy also limits the technologies of government, merely to use broader regulatory and policy measures. Addressing this gap in the literature, therefore, this paper examines the following research questions: (*i) What are the calculative technologies imposed in the COVID-19 response by the government of England? (ii) To what extent is the COVID-19 response as a set of calculative technologies different from what has happened before? (iii) What makes the governance of COVID-19 stand out regarding bio-politics*?and *(iv) Cross-referencing from other contexts, what are the implications of the application of calculative technologies? *

 The study uses Foucault’s governmentality and bio-political scholarship^15-18^ to analyse the UK government’s population governance strategies executed during the first wave of COVID-19. Foucault’s governmentality refers to the ways in which the state exercises control over, or governs, the body of its population. In his discussions on the art of government, Foucault specifically discusses ‘bio-politics’ as the way for neo-liberal governments to manage their populations and administer the mechanics of life, eg, reproduction, births and mortality, health quality, life expectancy and safety and security, through broader technologies of government, such as statistical analysis and controls, population level regulations and surveillance mechanisms. Chatterjee^19^ argues that some governments (eg, post-colonial) can even use these rationally manipulated bio-political techniques, instruments and policies to frame their citizens as ‘subjects’ and favour some population groups over others. These broader ‘technologies of government,’ in the more visible and distinguishing sense, are involved and connected with rational calculative practices: the ‘technologies and procedures’ through which conceptions of proper modes of governing populations are developed ie, risk assessment, cost/budget calculation, performance management and accountability.^20-23^ The use of ‘calculative techniques’^23,24^ as a controlling and surveillance mechanism in natural disaster situations, eg, earthquakes, hurricanes, bushfires, droughts and floods, is not a new topic and is discussed mainly in the disaster accounting literature.^25-32^ Such practices are central to governmentality and bio-politics during COVID-19, the application of which creates a particular form of ‘public visibility’ and ‘rationale’ to events and activities such as “*what is good, healthy, normal, virtuous, efficient or profitable*” (p. 175), and make the society governable at a distance,^21,23^ with numbers, standards and metrices in the lives of individuals and the functioning of society. The paper interprets these COVID-19 related self-governance reconfigurations enforced by the government as a bio-political intervention.

###  Setting: The COVID-19 Context in the United Kingdom

 As of July 21, 2021, a total of 5 723 422 infected cases and 129 446 deaths were reported in the United Kingdom, the highest death rate among European countries.^33^ The UK government received criticism at a global level for its response to COVID-19. For instance, a report by the Economist Intelligence Unit^34^ reveals that the UK responses were among the worst, revealing a low score of 2.2 out of 4. The index considered three calculative measures of the quality of responses (tests, provision of non-COVID healthcare, death rate) and calculative risk factors (obesity prevalence, share of population age 65+, international arrivals). The report claims that “an insufficiently fast and co-ordinated response, an initial lack of testing capacity, and a decision to suspend track and trace in early March explain why the United Kingdom became an outlier.”^34^

 As per the Global Health Security Index^35^, the United Kingdom was initially the second-best prepared country to respond to healthcare pandemics. The UK’s poor response to COVID-19 was therefore not anticipated. Public health experts claim that* “there’s a reason the scorecard got it so wrong: it did not account for the political context in which a national policy response to a pandemic is formulated and implemented*.*”*^36^ In addition to political reasons, compliance with World Health Organization (WHO) guidelines and calculative measures of ‘track and trace’ were poor in the United Kingdom. For instance, Dr. Jenny Harries, England’s Deputy Chief Medical Officer, commented on the relevance of WHO guidelines, stating, “*The clue with WHO is in its title—it’s a World Health Organization and it is addressing all countries across the world, with entirely different health infrastructures*.*”*^36^ Moreover, calculative practices such as the disproportionate impact of COVID infection ratesof Black, Asian, and Minority Ethnic (BAME) communities, the non-prioritising of healthcare workers, misunderstanding of the difference between ‘science based’ and ‘political based’ decisions, and over reliance on the traditional concept based ‘preparedness,’ characterised the UK’s poor response to the pandemic.^37^

## Methods

 The study sourced secondary documents from reliable sources including government publications, parliamentary Hansards, the National Health Service (NHS) publications, and national newspapers. More specifically, the study ensures that key narratives are derived from well-established scientific sources, responsible individuals and respective government institutions. For instance, the document review was followed by the analysis of quotes by politicians, government officials, NHS staff members, scientists, public health experts, health policy-makers, COVID-19 patients, the general public, the media, the business community and the WHO representatives. The data sources focused only on the first wave of government responses from March 2020 to December 2020. Additionally, the researchers observed events such as daily media conferences, parliamentary debates, government investigations, and political interventions that occurred during the same period.

 The study thus employs ‘critical thematic analysis’ to examine the interrelationships between the technologies of government and bio-politics.^38-40^ This approach helps identify the specific social construction of shared realities regarding the government’s bio-political projects. An open coding approach is used to develop the themes, having identified the experiences and perceptions of key actors in the published statements, media reports and public documents. The iterative process between data reduction and drawing and verifying conclusions conducted at various levels, eg, individual accounts, organisational and wider socio-political contexts, has proved useful in searching for the direct and indirect connections between calculative techniques and bio-politics. The analysis derived five major themes, namely (*i*) justifying the government’s intervention in daily lives through accounts and matrices; (*ii*) calculative instruments used for governance; (*iii*) disciplining and policing the public; (*iv*) demonstrating public accountability through prioritisation and categorisation; and (*v*) criticisms of the government’s calculative measures. The primary coding process was commenced by two co-authors. During the process, some evidence overlapped between the government’s intervention in daily lives through accounts and matrices and the calculative instruments used for governance. However, after several in-depth discussions, all co-authors agreed on the five themes, based on the contextual setting and the availability of secondary data. The authors recurrently reviewed the research design, analysis and interpretation to agree on the conceptual and empirical alignment of themes and data sources.

## Results

###  Numbers/Accounts and Matrices: The Rationales for Government Intervention in Public Lives

 During the first wave of COVID-19 in England, the UK government employed statistics on the death toll, the reproduction number (R), and resource statistics and narratives to frame the public discourse around COVID-19 and try to control spread of the virus. These numbers/statistics and matrices provided the ‘visibility’ for the daily events and crises faced by the public and also offered the rationale for the government’s undeviating interference in public lives during COVID-19.

 First, the total number of deaths from COVID-19 was used as a benchmark to assess and communicate the effectiveness of early responses. The United Kingdom had one of the highest COVID-19 death rates in the world. However, there were criticisms as to how different countries referred to different bases of reporting methods to count the death toll. The UK government was of the view that the comparison of COVID-19 deaths across countries therefore did not reflect reality. Dominic Raab, the UK’s foreign minister, commented on the different approaches:

*“There are different ways of counting deaths, as we know…we’ve had that debate in this country. We now publish data that includes all deaths in all settings, and not all countries do that. So, I’m not sure that the international comparisons work unless you reliably know that all countries are measuring in the same way*.*”*^41^

 The statistics on the death toll also created the visibility for public debate and criticism against the government. Using the death toll numbers and rationale, Professor Martin McKee from the London School of Hygiene and Tropical Medicine suggested that regardless of the differences between countries, the UK’s death toll rose mainly due to government delay in imposing lockdown:

*“It’s actually quite simple…if we look at the countries that responded quickly and got in at the very beginning, they’re the ones that have managed to contain the epidemic*.*”*^41^

 Second, reducing the coronavirus reproduction (R) number was treated and communicated to the public as one of the key rationales to determine the transmission of the virus. The importance of maintaining R below 1 was emphasised, so that each existing infection would cause less than one new infection and thus help control the spread of the virus. Health authorities were very careful about the R number as it was a crucial measurement that would decide on lockdown restrictions. Professor McKee explained the link between lockdown and the R number:

*“We typically think that one person infects between 2.5 and 3 other people … Say they do that every day, they’ll infect 3 then 9 then 27 and it goes up to about 20 000 in a matter of about 10 days*.* That’s the challenge: if you can get the R number down to 1.5 then you can reduce that number from 20 000 additional cases down to about 40. So even a few days makes a huge difference*.*”*^41^

 A low R number would have provided the government with the rationale to start easing the lockdown. Sticking to compliance with the lockdown was therefore urged. Commenting on the consequences of non-compliance with lockdown measures and its influence on the R number, Bournemouth East’s MP Tobias Ellwood remarked:

*“Let’s not forget the pandemic is far from over. Rules may be relaxing but restrictions remain in place for a reason…If they are ignored and the R value rises then tougher lockdown rules will return. Let’s stay alert to the dangers of COVID-19*.*”*^42^

###  Calculative Instruments Used for Governance at a Distance

 Similar to many other countries (see eg, New Zealand), the UK government also used various calculative instruments to control public behaviour, as a way of reducing infection rates.

*First*, the government announced ‘20 second hand washing with soap in warm water’ as a key healthcare principle that people should follow to prevent the spread of the virus and protect themselves. Hand washing was strongly advised after blowing the nose, sneezing, coughing, before having food, and immediately after returning home. In public places, people were advised to avoid touching their nose, eyes and mouth without hand washing. In the case of a cough or sneeze, the general public was asked to use a tissue or hand as appropriate, and clean their hands immediately afterwards.

*Second*, there was the practice of ‘maintaining a social distance of two metres’ to slow down the spread of the virus. In making the recommendation, science was referred to, based on the claim that respiratory droplets can travel up to 2 metres. The Centre for Disease Control and Prevention provided more clarity on social distancing: “*staying away from mass gatherings and keeping a distance of 6 feet or 2 metres – about one body length – away from other people*.”^43^ Thomas Perls, Professor of Medicine, Boston University, commented on the significance of the two-metre distance regulation:

*“It limits the number of people an infected person comes into contact with – and potentially spreads the virus to – before they even realize they have it.”* ^43^

 Dr. Robin Thompson, Junior Research Fellow in Mathematical Epidemiology at the University of Oxford, referred to this calculative instrument of 2 metre social distancing as the reason for reduced transmission of the virus (33% fewer contacts) from 1093 (without social distancing) to 127 cases within six weeks.^44^ Health authorities also used this instrument of ‘complying with the social distancing regulations’ as the basis for demanding that individuals play a critical role in substantially reducing the spread of virus. The failure to adhere to the ‘1-metre-plus’ instrument was also used by the health authorities to rationalise and communicate their healthcare resource issues to the public. It was announced publicly that the healthcare facilities to manage the number of cases, known as *flattening the curve,* was challenging.

*“If the number of cases isn’t kept below what the healthcare system can handle at any one time – called flattening the curve – hospitals could become overwhelmed, leading to unnecessary deaths and suffering*.*”*^43^

 Using the ‘level of the spread of the virus’ as a calculative measure, later the government also prohibited public gatherings of more than two family units. Group gatherings were limited to 6 members. In order to make the public more disciplined and controlled, the government announced that those who did not comply with these requirements were subject to fines.

*Third*, ‘self-isolation’ (quarantine) was a key calculative instrument of the UK government’s strategy to minimize the spread of the virus. The scientifically chosen numbers/statistics and narratives were added to this instrument for creating public acceptance and visibility. For individuals showing symptoms, health authorities advised them to self-isolate for seven days from the day symptoms started. After contact with an infected person, household members were required to stay at home for 14 days. Individuals with underlying medical conditions were advised, if possible, to take extra steps to protect themselves (shielding). Individuals with symptoms were not allowed to attend GP surgeries, pharmacies or hospitals. Individuals were requested to use the NHS online service or make a call only for urgent medical help. Referring to the calculative instrument of self-isolation, the UK Prime Minister Boris Johnson advised the general public to self-isolate after being infected by the virus:

*“…on the advice of the Chief Medical Officer, I’ve taken a test that has come out positive, so I am working from home. I’m self-isolating and that’s entirely the right thing to do... So, thank you to everybody who’s doing what I’m doing, working from home to stop the spread of the virus from household to household*.*”*^45^

*Fourth*, with the number of infections increasing, the government then added another phrase to the ‘social distancing’ instrument, to ‘restrict outdoor exercise.’ Considering the limited access to parks and back gardens, the government decided to allow one hour, outdoor workouts per day for the general public. This newly introduced ‘outdoor restrictions’ instrument, however, received some criticisms from the public. Linda Bauld, Professor of Public Health at the University of Edinburgh, commented on the severe and increasingly complex implications due to restricted outdoor activities:

*“The health implications of the lockdown that we anticipate – increased alcohol consumption, domestic violence, anxiety and depression, poor diet and decreased physical activity - will get worse if we confine more of us to our homes, without the hugely important respite that outdoor exercise provides*.*”*^46^

###  Disciplining and Punishing the Public Through Performance Measures and Controls 

 In order to create self-governable and comparable individuals, the government imposed strict behavioural control measures, such as lockdown compliance and non-compliance fines for the public.

*First*, by declaring a national emergency, the government instructed the public to ‘stay at home’ and restrict their everyday movements, with four exceptions: infrequent shopping for basic needs (eg, food and medicine), one hour, outdoor workouts, supporting vulnerable people, and for travel to and from work where the job could not be done from home. Except for members of the same household, public gatherings of more than two people were banned. While funerals were allowed with limited participation, all wedding ceremonies and sports events were cancelled. While government guidelines were necessary to reduce the spread of the virus, some people felt that it was quite challenging to adhere to these government imposed ‘behavioural controls’ in everyday life due to various practical issues, such as economic and psychological breakdowns. For instance, a pharmacist commented that:

*“I was working in a pharmacy yesterday and a member of staff broke down. Same happened last week: I was working in a pharmacy, I had to make a member of staff a cup of tea because they’d broken down. They just cannot cope*…*We need tighter guidance on what constitutes ‘essential’ and enforcement measures*.”^47^

*Second*, the government decided to impose a minimum £30 fine on those who did not respect and comply with lockdown rules and warned that this would rise significantly for further offences.^42^ The BBC reported that as per the The National Police Chiefs’ Council figures, 15 552 fines had been recorded by May 29, 2020 in violation of social distancing regulations. The government instructed the police to employ a four-step systematic approach (*Engage. Explain. Encourage. Enforce)* to make people understand the need for the regulations and to maintain control of the situation.^48^ The failure to pay the fine resulted in legal consequences with court involvement and ultimately, payment of the fine. For instance, in England, people aged 18 or above were fined £100 for the first offence, reduced to £50 if paid within two weeks. The second offence would be £200, doubling for subsequent offences to a maximum of £3200. Vikki Slade, the Liberal Democrat leader of Bournemouth Council, commented that regardless of the various efforts taken by the council, people did not comply with the rules and did not respect the community.

*“It doesn’t matter what we do, these vile idiots will ignore rules … disgusting*.*”*^49^

 For instance, the Cheltenham festival was criticised by some who claimed that the event would become an epicentre of the pandemic. Around 150 000 people took part in the four-day event ten days before the lockdown. These results demonstrate the underlying difficulties of using ‘authoritarian’ behavioural controls to govern people’s everyday lives.

###  Construction of “Public Accountability” Through Prioritisation and Categorisation 

 Similar to other countries,^50^ the government thus used some carefully chosen ‘calculative measures’ such as prioritization and categorization of healthcare staff and clinically extremely vulnerable individuals for preferential treatment, to demonstrate their moral responsibility and ‘accountability to the public.’ The government used the calculative technology of ‘risk assessment’ to rationally identify and group these people.

*First*, the government defined and prioritiseda certain number of professions as key workers during the pandemic, including frontline NHS staff, all healthcare professionals (eg, pharmacists), teachers and those who were engaged in delivering essential services. Key workers were put into priority groups. For instance, large stores announced dedicated hours for NHS staff. In addition, key workers were prioritised for polymerase chain reaction (PCR) testing, could send their children to school, ask for discounts, and apply for a driving licence, to name a few. Highlighting the importance of prioritising the key workers, Ruth Rankine, the director of primary care at NHS Confederation, commented:

*“Many frontline staff are ‘really, really scared’…When they go in and see a patient... even though they’re not displaying symptoms, they may still have the virus…We are seeing increasing numbers of primary care workforce going off sick as a result*.”^51^

*Second*, in order to shield vulnerable individuals, the NHS categorised them according to two levels of higher risk that would require extra attention during the pandemic. The high-risk group included clinically extremely vulnerable individuals with underlying medical conditions (eg, over 80 years). The moderate risk group included clinically vulnerable individuals (eg, above 70 years). Termed as ‘shielding,’ vulnerable individuals were then strongly advised with some specifically targeted ‘calculative measures,’ such as to stay at home whenever possible, keep outside visitors to a minimum and strictly comply with the healthcare guidelines. Further, the clinically extremely vulnerable individuals were advised not to be a part of the ‘support bubbles’ which were created among other households.

###  Criticisms of the UK Government’s Use of Calculative Technologies 

 In several instances, the UK government’s COVID-19 policies made visible to the public through calculative technologies encountered challenges in their implementation. This resulted in growing public criticism of the government’s handling of COVID-19.

*First*, the Prime Minister and his ministers were criticised and questioned over a lack of clarity from the government with regard to the COVID-19 guidelines, including social distancing instruments and measures. For instance, the BBC reported contradictory views expressed by the government: “only jog for 30 minutes,” “children should not travel between parents,” “you can shop once a week,” “people should leave the house once, if possible.”^52^ Moreover, there were criticisms against public office holders for not complying with the social distancing rules. For instance, the former Cabinet Secretary, Gus O’Donnell, urged politicians to set an example to the public by complying with the regulations:

*“They do need to learn a lesson from this and actually obey their own rules much more strictly*.*”*^52^

 The media also reported how council workers were being physically and verbally abused while implementing the lockdown restrictions, because of the lack of transparency and public awareness on some of its guidlines.^47^ “*Workers have been spat at, sworn at and racially abused.”*^47^

*Second*, the government was also criticised for not adopting specifically targeted calculative measures to protect vulnerable groups in care homes where the majority of deaths were recorded. Responding to a question about this, the Prime Minister claimed that:

*“Too many care homes didn’t really follow the ‘procedures’ in the way that they could have*.*”*^53^

 The National Care Forum commented that these allegations were “*frankly hugely insulting … clumsy and cowardly.*”^54^ Labour’s shadow health secretary, Jonathan Ashworth, highlighted several shortcomings in calculative technologies of COVID-19 that led to the high death toll in care homes:

*“Care providers were sent conflicting guidance throughout this outbreak, staff could not access testing until mid-April and are still not tested routinely, PPE [personal protective equipment] supplies have been inadequate, thousands of families have lost their loved ones in care homes to this disease, care workers themselves have died on the front line*.*”*^54^

*Third*, the numbers produced by calculations, eg, COVID-19 risk analysis and cluster analysis of infected people in the population, indicated existing healthcare inequalities in the United Kingdom. Particularly, the BAME community appeared to be particularly vulnerable and ‘disproportionately’ affected by COVID-19 mortality and morbidity rates. The public health review reports that the highest age standardised infection rates per 100 000 people were among BAME communities (486 in females and 649 in males) and the lowest was among white ethnic groups (220 in females and 224 in males). People of Bangladeshi ethnicity were reported as twice as much at risk of death than people with British white ethnicity. At the same time, other ethnicities such as Chinese, Pakistani, Indian, and other individuals with Asian, Caribbean and African backgrounds also reported a 10%-50% higher death risk compared with British white individuals. The number of deaths among NHS-BAME staff was widely discussed, with several explanations being given. The BBC highlighted key underlying factors:

*“The unequal impact may be explained by social and economic inequalities, racism, discrimination and stigma, differing risks at work and inequalities in the prevalence of conditions such as obesity, diabetes, hypertension and asthma, which can increase the severity of Covid-19*.*”*^55^

 In particular, the PHE review report reveals that historical racism largely prevents BAME staff members seeking PPE and necessary healthcare services when needed.

*“…historical racism may make BAME individuals less likely to seek care when needed or, as NHS staff, to speak up when they have concerns about PPE or increased risk*.*”*^56^

 This signified the unequal results of COVID-19 governance in the United Kingdom, as some citizens are better at utilizing their agency for demanding public goods, and that marginalised others are less willing or able to do so, because of different socio-economic factors. Rehana Azam, GMB National Secretary commented that:

*“Equally obviously, structural inequality could ultimately be to blame. If an ethnic group is at higher risk because of overcrowded housing, deprivation or poorer general health rather than strictly race, that is still a finding that many people will find troubling*.*”*^57^

 In response to these public criticisms, the NHS introduced a new calculative technology for healthcare workers, designated as ‘COVID-19 Risk Reduction Framework.’ The framework consisted of workplace, workforce and individual assessment criteria, measures and instruments. Four underlying factors were particularly considered in individual risk assessments: age and ethnicity, sex, underlying health conditions and pregnancy.

## Discussion

 The results/findings above demonstrate the role that calculative technologies^20-23^ played in the COVID-19 responses by the government of England and how they made the governance of COVID-19 stand out with regard to bio-politics. While pandemics such as COVID-19 are not common, the way the governments give them meaning through ‘calculative practices’ have been challenging and ongoing. Thus, findings of this study demonstrate how the UK government forced their citizens to pursue prescribed and often standardized targets, calculative instruments and measures in their everyday lives and in so doing, they were brought under the regime of governmentality.^58-61^ These calculative measures and instruments, eg, 2 metre social distancing, restriction to their everyday life movements and the imposition of fines on those who did not comply with public rules made the public self-responsible for mitigating the risk of COVID-19 infection, and then subsequently safeguard their living. These bio-political initiatives made the government widen their base by which citizens’ life was governed at a distance, and so, successfully gained bio-power over its citizens.^15-18^ These were socially marketed as kinds of obligations and a way to protect themselves and vulnerable groups during the pandemic.^55,59^ However, COVID-19 also shed light on existing healthcare inequalities, even though the UK government attempted to communicate their public accountability via some calculative measures such as prioritisation and categorisation of healthcare staff and vulnerable people for preferential treatments. The calculative analysis such as risk measurement thus indicated that life-prolonging decisions have only been possible or available for certain citizens, whereas in others, eg, BAME, the COVID-19 responses have not treated all citizens equally.

 This new and temporary environment inevitably created by COVID-19 thus emerged as a key site for bio-politics, involving obligations, entitlements across different fields and scales of engagement by the public.^19^ As results/findings suggest, this UK government’s approach of governing at a distance increasingly considers the individual as an autonomous agent (subject) who self-monitors and exercises his/her agency in order to mitigate external risks.^15-18^ The logics of ‘countability’ and ‘measurement,’ eg, comparative death tolls, infection rates and coronavirus R (Reproduction) number, lockdown rules such as one hour outdoor workout, limited participation in events have been used as the tell-tale ‘bio-political’ mechanisms of life management and governmentality. In other words, these self-governing calculative technologies enacted by the government created some self-disciplined limits and also police-administered ‘punishments,’ eg, fines to lockdown rule breakers, with the promise of safe-guarding people’s lives.^15-18,21,23^ Thus, citizens were made privately accountable to themselves (self) and also publicly, to their families and fellow community members. By using these widely communicated numbers/matrices, calculative measures and instruments, the UK government created a bio-politically driven public discourse during COVID-19 that ‘life can be managed through calculations.’

## Conclusion

 This paper examined how the government of England deployed calculative technologies to govern their citizens, particularly during the first wave of COVID-19. Foucauldian literature on COVID-19 in other countries,^5-10^ and the general COVID-19 literature in the United Kingdom,^1-4^ forgets the importance of explicitly and distinctively used calculative technologies in COVID-19 governance. Instead, their works largely refer to broader technologies of government and also bio-political analysis of COVID-19. In particular, the studies beyond the UK address the complex set of ‘liberal’ and/or ‘coercive’ technologies that are used to rationalize the political power of governments, but without an explicit and distinguishing explanation of the role and importance of calculative technologies.^6,7^ In contrast, most of the UK studies focus on the managerial and decision-making aspects of COVID-19, without an emphasis on bio-political implications.^3^ Therefore, researchers should take note that an important empirical contribution could be to implement ‘calculative’ researches to avoid such a potential mistake, and consider this approach as a significant part of government technology, as detailed in this paper.

 The paper also elaborated on the transformative nature of calculative technologies. Thus, the set of calculative technologies used in the COVID-19 response in the United Kingdom produced an incrementally changed approach from the past health emergency governance systems, eg, the NHS in the United Kingdom. This new system that attempted to deal with the temporal environment created by COVID-19 adopted a tailor-made approach to target and prioritise specific population categories. Also, this ‘temporal environment’ required more self-governance principles for citizens compared to the past liberal governance rules; the COVID-19 governance in the United Kingdom stands out very much regarding the bio-politics implemented through authoritarian principles: self-discipline and punishment. Regarding future implications, when cross-referencing with other contexts, the government must focus more on introducing a set of calculative technologies within their broader technologies of government, specifically targeting vulnerable communities, eg, ethnic minority groups, in order to create better social justice and public accountability. The study’s findings also suggest that the UK government must attempt to enact a better ‘health and recovery’ environment, eg, adequate funding and resources for the NHS, rather than focusing on its self-governance strategies and political controls over its citizens as ‘subjects.’^19^

## Ethical issues

 This study uses the freely available data on the Internet, books or other public forums. Therefore, the permission for further use and analysis is implied/allowed under the UK’s Data Protection Act (2018).

## Competing interests

 Authors declare that they have no competing interests.

## Authors’ contributions

 Conception and design: KJ and TJ. Data collection: TJ and CW. Analysis and interpretation of data: CW and KJ. Drafting of the manuscript: KJ, TJ, CW, and PA. Critical comments and editorial review: KJ and PA.

## Disclaimer

 This paper represents the opinions of the authors, and is the product of academic research.

## Funding

 The author(s) received no financial support for the research, authorship, and/or publication of this article.
